# Reduction of Excessive Dietary Sodium Consumption: Effectiveness of a Prevention Intervention among Health Workers in a Large Italian Hospital

**DOI:** 10.3390/ijerph20085478

**Published:** 2023-04-12

**Authors:** Gianluca Spiteri, Maria Grazia Lourdes Monaco, Angela Carta, Lorena Torroni, Francesco Taus, Giuseppe Verlato, Stefano Porru

**Affiliations:** 1 Occupational Medicine Unit, University Hospital of Verona, 37134 Verona, Italy; 2 Section of Occupational Health, Department of Diagnostics and Public Health, University of Verona, 37134 Verona, Italy; 3 Section of Epidemiology and Medical Statistics, Department of Diagnostics and Public Health, University of Verona, 37134 Verona, Italy; 4 Unit of Forensic Medicine, Department of Diagnostics and Public Health, University of Verona, 37134 Verona, Italy

**Keywords:** salt consumption, health promotion, health prevention, occupational medicine, workplace educational intervention

## Abstract

Excessive salt consumption is one of the leading causes of high blood pressure. Worldwide salt intake largely exceeds the WHO recommended amount. This study aimed to evaluate the prevalence of high salt consumers and the effectiveness of a short-term workplace educational intervention among health workers. An online survey, assessing daily salt consumption through the MINISAL-SIIA questionnaire, was sent to the 4911 health workers employed by the University Hospital of Verona, Italy. Health workers who had a high (total score ≥ 10) or moderate (total score = 8/9) salt consumption associated with obesity or arterial hypertension were invited to undergo a medical examination and a short individual counselling session. A total of 1665 health workers (34.0%) completed the online questionnaire; 40.9% and 12.6% had moderate and high salt intake, respectively. High salt intake was more prevalent in men, current and past smokers, and obese and overweight subjects. In 95 participants completing the clinical phase, median daily salt consumption decreased from 10 (p25–p75 8–11) to 7 g (6–8) (*p* < 0.001), systolic blood pressure from 130 (120–140) to 120 (120–130) mmHg and weight from 78 (62–87) to 75 (62–86) kg. More than half of health workers had an excessive salt intake. However, a brief educational intervention in the healthcare working setting can substantially reduce unhealthy dietary habits, fostering weight loss and blood pressure control. Studies with a longer follow-up are needed to evaluate the persistence over time of these effects.

## 1. Introduction

Excessive salt consumption is one of the leading causes of high blood pressure, which, in turn, is the main risk factor for cardiovascular diseases [1]. Several studies have shown it is also related to a greater risk of kidney disease and stomach cancer [2]. Furthermore, deaths and Disability-Adjusted Life Years related to excessive salt consumption globally increased in the last 30 years, peaking at 1.89 million and 44.87 million, respectively, in 2019 [2]. Lastly, an association between high salt consumption, higher BMI and greater waist circumference was well described by several studies [3,4,5].

WHO recommends a dietary salt intake of less than 5 g/day, corresponding to an amount lower than 2 g/day of sodium [6].

The Global Burden Study 2010 revealed a mean worldwide salt intake of 10 g daily. In 181 out of 187 countries, the daily salt consumption was at least 2.5 g per day higher than the recommended and in 51 of them was more than 10 g per day [7]. These data clearly show that global action is needed to reduce salt consumption. A recent review, including 13 studies from 9 high-income countries and 4 low- and middle-income ones, investigated global salt consumption. Compared to the Global Burden Study 2010 results, a lower salt intake was found in Canada, Barbados, England and Italy. Conversely, a higher intake was detected in three low/middle-income countries, suggesting that significant differences in daily salt consumption persist in different geographic areas and that these gaps are increasing over time [7].

Furthermore, the Global Burden Study 2019 reported that sodium intake persisted unchanged in men and dropped slightly in women between 1990 and 2019, showing high variability between different geographical regions [2].

Donfrancesco et al. investigated the trend in the Italian adult population’s salt intake from 2008 to 2019. Consumption diminished both in men and women from 10.8 g/day to 9.5 and 8.3 g/day to 7.2, respectively, showing a more than 10% reduction in both categories. A similar trend was observed in other countries, such as Slovenia, the UK, Denmark, France and Ireland [4].

The interdisciplinary Working Group for Reduction of Salt Intake of the Ministry of Health (MINISAL-GIRCSI study) studied salt consumption among hypertensive patients in Italy, finding an excessive intake both in men (median 10.1 g/day) and women (median 8.1 g/day) (90.5% and 81.4%, respectively) [3].

Knowledge about the risk of excessive salt consumption significantly affects sodium intake. Idelson et al. administered a questionnaire on salt-health-related knowledge to 11,618 Italian citizens. They reported widespread awareness about this topic, but there were considerable differences in sociodemographic characteristics, suggesting that educational campaigns could be helpful [8].

A recent systematic review by Khalesi et al. evaluated the effectiveness of behavioural change interventions in adults. A reduction above one g/day was reported, proving that preventive actions, including individual consultation, educational sessions, reinforcements and food recording, help reduce salt consumption [9].

The most common method to evaluate the daily salt intake is 24-h urinary sodium excretion. To simplify the assessment, D’Elia et al. validated a 5-item questionnaire which showed a linear association between the score and urinary sodium excretion, allowing subjects to be detected with a high (score > 10) or very high (score > 13) salt intake, feasible in health promotion and rehabilitation studies [10].

The workplace represents an ideal setting for health promotion and dietary-oriented programs [11,12,13]. Beer-Borst et al. performed a study to evaluate a one-year workplace intervention to reduce salt intake in a Swiss working population, combining educational and environmental interventions. Among the 145 participants, the mean reduction was 0.6 g/day, associated with decreased blood pressure [14,15]. Even better results were achieved in a Japanese study which compared the salt intake in a manufacturing company (Company A) to a construction company working population (Company B) (69 and 68 subjects, respectively). In company A, the authors implemented a healthy intervention which included healthy lunch and nutrition education. Workers of Company B were observed as a comparison. One year after the start of the study, the mean salt consumption in company A decreased from 10.7 to 9.3 g/day, while no change was observed in company B [16].

Health workers (HW) are at high risk for an unbalanced diet, mainly due to shift working, prolonged working hours and work-related stress [17,18]. As a result, these factors can affect eating behaviours, increasing the consumption of sweets, snacks and sweetened beverages, as well as reducing the intake of vegetables and fruits [19,20]. This working population is, therefore, an ideal target for dietary improvement campaigns. However, to our knowledge, no studies have investigated the effectiveness of dietary educational programs in a large population of HW.

This study aimed to assess the following:

- The prevalence of subjects who report a low, moderate or high salt intake in a large HW population in Northern Italy;
- The effectiveness of a short-term workplace nutritional education intervention addressed to HW at high risk for salt-related diseases through subjective and objective indicators.

## 2. Materials and Methods

### 2.1. Study Design, Setting and Participants

A pre- and post-intervention study was carried out from January 2020 to January 2021 by the Occupational Medicine Unit and the Prevention and Protection Service of the University Hospital of Verona.

The research was addressed to 4911 HW, employed in the University Hospital of Verona at enrolling time and listed in the Prevention and Protection Service register. The study included an online screening (phase I), where high-risk individuals were identified and invited to undergo a clinical evaluation associated with an educational intervention (which represented phase II).

Ethical approval was received from the “Comitato Etico per la Sperimentazione Clinica delle province di Verona e Rovigo” (Prot. N. 56297 14 October 2019). Written informed consent was obtained from all subjects at phase I.

### 2.2. Phase I

A structured questionnaire was sent to the institutional email addresses of the University Hospital of Verona employees. The questionnaire included sociodemographic items (sex, age, date of birth, educational level, job title) and clinical information (smoking habits, ongoing therapy for arterial hypertension), and a five-question validated questionnaire for the evaluation of daily salt intake (MINISAL-SIIA) [10]. While answering the questionnaire, subjects were also asked to release informed consent. A score from 1 to 3 was assigned to each of the five questions so that the total score ranged between 5 and 15 [10], and subjects were divided into three categories:

- Adequate salt intake (total score < 8);
- Moderate salt intake (total score = 8/9);
- High salt intake (total score ≥ 10).

HW in the high salt intake or moderate category, associated with obesity or treated arterial hypertension, were considered at increased risk for salt-related diseases.

### 2.3. Phase II

HW at high risk for salt-related diseases were invited to undergo a medical examination, including assessment of blood pressure, height, weight, and hip and waist circumference according to national and international guidelines [21,22,23].

A Bioelectrical Impedance Analysis was performed using BIA 101 ANNIVERSARY Sport edition (Akern) to assess resistance (Rz), reactance (Xc) and phase angle. Fat mass and free fat mass were estimated by Bodygram PLUS software (Akern).

Following the clinical and instrumental examination, trained medical staff conducted an individual counselling session of 15 min about risks related to excessive salt consumption, foods with higher salt content and appropriate behaviours to reduce salt intake.

A copy of the brochure “WORLD WEEK 2019—Less salt more health”, edited by World Action on Salt & Health and the Italian Society of Human Nutrition, was given to each participant (Italian version available at https://eng.sinu.it/meno-sale-piu-salute/, accessed on 15 March 2023).

A follow-up examination was performed 4–6 months after the first one. Blood pressure evaluation, hip and waist circumference measurement, and BIA were remeasured. Participants were asked to fill out again the questionnaire about salt consumption.

### 2.4. Statistical Analysis

The values measured before and after the intervention were presented using appropriate descriptive statistics. Qualitative variables were reported using absolute and percentage frequency tables; quantitative variables with asymmetric distribution through median and interquartile range (IQR); and quantitative variables with symmetric distribution by means and standard deviation. Prevalence estimates were presented with a relative 95% confidence interval. The associations between salt consumption (coded as adequate, moderate and high consumption) and potential determinants (sex, age group, profession) were studied by non-parametric Kruskal–Wallis test for quantitative variables and Pearson’s chi-squared for categorical variables. A multinomial logistic model further evaluated determinants of salt intake. In particular, salt intake (adequate, moderate, high) was the response variable and sex, age, BMI (normal-weight, overweight, obese), job title (nurse/physician/technician/administrative/other) and smoking habits (never, former, current smokers) the potential determinants.

For pairwise comparisons, significant differences were determined by the Wilcoxon signed-rank test for quantitative and ordinal variables.

Analysis was performed using STATA statistical software, release 17 (StataCorp, College Station, TX, USA) and statistical significance was set at *p* < 0.05.

## 3. Results

**Figure 1:**
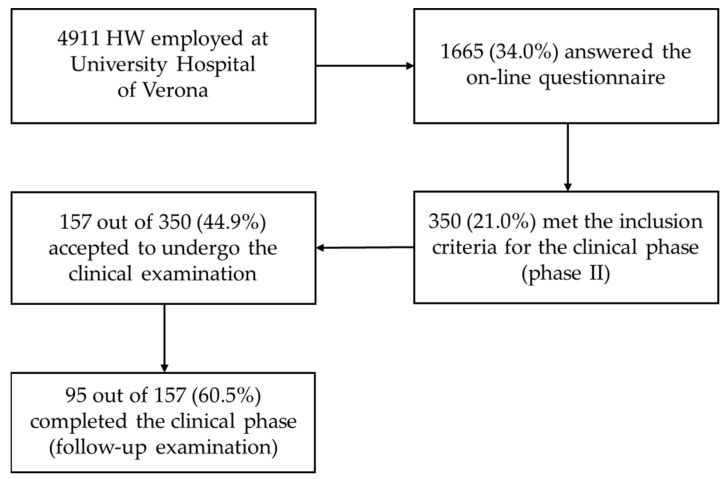
Flow diagram from enrolment to the conclusion of the study.

Of 4911 HW, 1665 (34.0%) completed the online questionnaire (Figure 1). As shown in Table 1, responders comprised a more significant proportion of women born in Northern Italy and nurses, and a lower proportion of physicians than non-responders.

### 3.1. Phase 1: Online Screening

The numbers of HW with adequate, moderate and high salt intake were 775 (46.5%), 681 (40.9%) and 209 (12.6%), respectively (Table 2). HW with higher than adequate salt intake had a higher prevalence of men and current/former smokers, higher BMI and a higher proportion of people born in Southern Italy or abroad. On the other hand, salt intake did not vary as a function of age, education level, job title, working seniority or drug-treated hypertension.

Age, BMI, job title and smoking habits emerged as the main determinants of high salt intake (Table 3). The risk of high salt intake decreased with advancing age, increased in overweight subjects with respect to normal-weight ones and nearly doubled in obese ones. In addition, the risk of high salt intake was the highest among technicians and administrative staff. As regards smoking habits, former smokers were at higher risk of high salt intake than never-smokers. Male sex was significantly associated with moderate salt intake.

### 3.2. Phase 2: Clinical Intervention in High-Risk HW

Out of 1665 responders, 355 HW (21.0%) met the criteria for inclusion in the clinical phase of the study. Their sociodemographic characteristics are reported in Supplementary Table S1. One hundred fifty-seven of them (44.9%) accepted to undergo the clinical examination; only 95 (60.5%) completed the follow-up clinical phase.

**Figure 2:**
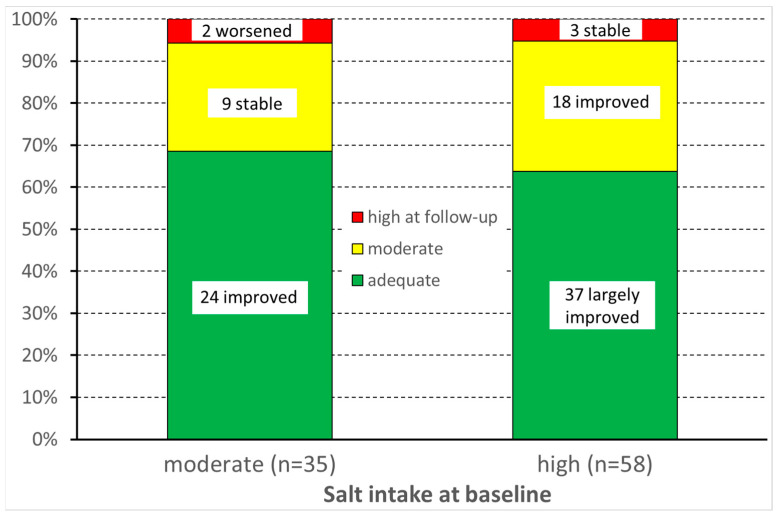
Variation in salt intake before and after the intervention.

Among them, the median score in the MINISAL survey significantly decreased between baseline examination and follow-up from 10 (p25–p75 = 8–11) to 7 (6–8; *p* < 0.001). At the individual level, salt intake decreased in 69% and 92% of HW with moderate and high salt intake, respectively. It increased in only two HW (8%) with moderate salt intake at baseline (Figure 2).

Table 4 reports the answers to single items of the dietary salt intake questionnaire given by the 95 participants who completed the clinical phase of the study. The consumption of salt, salted bread and cheese/cold cuts significantly decreased after the educational intervention. Moreover, HW became less likely to get thirsty after a meal and more prone to perceive the food as salty.

Table 5 shows anthropometric indexes and bioelectrical impedance values measured before and after the intervention. Median weight decreased by 3 kg after the educational intervention and abdominal circumference by 1 cm. Consequently, BMI and waist-to-hip ratio significantly decreased, while fat-free mass slightly increased. Remarkable improvements in arterial pressure were also observed, as median systolic and diastolic pressures decreased by 10 and 5 mmHg, respectively.

## 4. Discussion

The online questionnaire was sent to the entire working population of the University Hospital of Verona. Even if low (34%), the response rate was more than twice that observed in another mailed survey (15.5%) addressed to a similar-sized HW population (4980 nurses) [24].

Significant differences in response rates were recorded among participants. Females answered the questionnaire more frequently than men, as did nurses than doctors. These data seem to underline the greater attention of females to health issues, according to the results found in a previous study [25]. In the same way, people born in Northern Italy were more likely to participate than people born in Southern Italy or outside Italy. No relevant differences were found among other job categories and age (Table 1).

The online survey showed that 40.9% and 12.6% had moderate and high daily salt consumption, respectively. These values were much lower than those recorded among hypertensive patients (86% high salt consumers) [10] and the general population. Indeed, 97% of Italian men and 87% of Italian women presented a salt intake, assessed by 24-h urine collection, above the WHO recommendations [4]. Similar results were reported in the MINISAL-SIIA study, in which excessive salt consumption was disclosed in 90% of men and 81% of women [3]. This difference could be explained, at least partly, by the target population of the present study, focused on HW, who have a greater awareness of the risk of an unbalanced diet than the general population [26].

High salt consumption was more frequent in males than females, in agreement with previous studies [4,10]. Median BMI significantly increased from HW with low salt consumption to those with high salt intake (22.7 to 24.5). Accordingly, a systematic review by Moosavian et al. found an association between higher salt consumption and greater BMI, which was attributed to increased sugar-sweetened beverages and energy intake [5]. Endocrine regulatory systems, such as leptin plasma concentration, could also be involved [5]. As a result, reducing salt intake may favour weight loss, increasing the benefits of the intervention.

The site of birth also reported significant differences in questionnaire scores. HW born in Southern Italy/Italian islands and outside Italy had a higher percentage of high salt consumers than those born in Northern-Central Italy. Accordingly, Idelson et al. [8] found a lower salt intake and greater adherence to the Mediterranean diet among southern Italy citizens, confirming that salt consumption varies among countries and across regions of the same country.

The educational intervention performed in the presented study achieved very high effectiveness in reducing reported salt consumption. Salt intake decreased in nearly all subjects (79 out of 93 = 85%) and reached an adequate level in 61 (66%). The improvement involved all the aspects investigated by the MINISAL-SIIA questionnaire, i.e., using salt at the table, consuming salted bread, eating cheese and cold cuts, feeling thirsty after meals and perceiving the food as salty. As a side effect, anthropometric indexes (weight, BMI, abdomen and hip circumference) and arterial pressure also significantly improved after the intervention. Absolute variations were low, probably due to the short follow-up period (4–6 months). However, the change in arterial pressure, amounting to −10 mmHg for systolic and −5 mmHg for diastolic pressure, was larger than the pooled estimates computed by the meta-analysis by He et al., amounting to −4.18 mmHg for systolic and −2.06 mmHg for diastolic pressure. The meta-analysis included 30 studies lasting from 4 weeks to 3 years [27]. In addition, a meta-analysis by Huang et al. found no association between the duration of the dietary intervention and the amount of blood pressure reduction [28]. Our results seem to confirm that the effect of dietary salt restriction on blood pressure begins in the first few months.

On the other hand, indexes derived by bioelectrical impedance were not significantly affected by the dietary salt restriction, except for a decrease in fat-free mass. Most changes in body composition and nutritional status in participants undergoing the nutritional intervention were too small to be detected by bioelectrical impedance [29]. To date, this method has been used mainly for evaluating the effectiveness of training programs in athletes and monitoring the nutritional status of critically ill patients [30,31]. Results reported in these studies suggest that bioelectrical impedance could also be helpful in dietary intervention contexts. However, a longer follow-up would be needed to achieve detectable changes in bioelectrical impedance indexes [32].

Health prevention projects, such as the present educational intervention, are considered cost-effective, as shown by the systematic review by Silva-Santos et al. [33]. Indeed, the authors found that, combined with other strategies, individual educational interventions effectively reduced daily salt intake (from 0.9 to 4.7 g/day) in the workplace [15,16]. A review by Geaney et al. also reported that workplace dietary modification interventions could improve workers’ eating habits, although long-term effects are still unclear [11]. A cost-effectiveness analysis of workplace nutritional interventions was carried out by Fitzgerald et al. [12], who found that the incremental cost-effectiveness ratio was under the Irish accepted ceiling ratio, so they can be considered cost-effective.

Our study has some limitations but also several strengths. The main limitation was the low adherence to the follow-up examination. Unfortunately, this phase was set up concurrently with the outbreak of the COVID-19 pandemic, during which it was impossible to recruit participants. However, non-response bias tends to affect prevalence estimates but not estimates of association [34]. Anyway, a preliminary information campaign about the study’s aims could help improve the adhesion. Another major limitation was that the daily salt consumption was evaluated through a validated self-administered questionnaire.

In contrast, most studies on dietary salt intake used urine collections and directly measured electrolyte intake. Although prone to information bias, we considered that this choice would involve a greater number of subjects. Moreover, urinary sodium assessment is expensive and affected by high variability, even with a constant dietary intake [10].

On the other hand, the participation in the online survey involved over 1600 HW. Therefore, to our knowledge, this study is the largest one evaluating the prevalence of high salt consumption in a healthcare working setting. Lastly, no previous studies used bioimpedance to evaluate the effectiveness of dietary salt restriction.

## 5. Conclusions

Our study showed a higher prevalence of low salt consumers among HW than the general population. A brief educational intervention in the health working setting demonstrated high effectiveness in reducing daily salt intake, blood pressure and weight in high salt consumers and in subjects who have a moderate consumption associated with obesity or arterial hypertension. Studies with a longer follow-up are needed to evaluate the persistence of lower salt dietary consumption over time.

## Figures and Tables

**Table 1:** Sociodemographic characteristics of health workers who did or did not respond to the online survey.

	Non-Responders *n* = 3246 (%)	Responders *n* = 1665 (%)	*p* Value
Sex			<0.001
Female	2210 (68.1)	1325 (79.6)	
Male	1036 (31.9)	340 (20.4)	
Age (median, p25–p75)	51 (42–57)	50 (43–55)	0.092
Geographical area of birth			<0.001
North Italy	2651 (81.7)	1436 (86.2)	
Centre	75 (2.3)	42 (2.5)	
South/Isles	408 (12.6)	139 (8.4)	
Abroad	112 (3.4)	48 (2.9)	
Job title			<0.001
Nurse	1261 (38.8)	751 (45.1)	
Physician	634 (19.5)	223 (13.4)	
Technician	416 (12.8)	204 (12.3)	
Administrative	373 (11.5)	199 (11.9)	
Other HW	562 (17.3)	288 (17.3)	

*p*-values were computed using Pearson’s chi-squared test and considered statistically significant if *p* < 0.05.

**Table 2:** Comparison among HW found with adequate, moderate and high salt intake via the online survey.

	Adequate Salt Intake *n* = 775 (46.5%)	Moderate Salt Intake *n* = 681 (40.9%)	High Salt Intake *n* = 209 (12.6%)	*p* Value
Sex				0.002
Female	646 (83.4)	519 (76.2)	160 (76.7)	
Male	129 (16.6)	162 (23.8)	49 (23.3)	
Age (median, p25–p75)	50 (43–55)	50 (43–56)	49 (42–55)	0.641
BMI, kg/m^2^ (median, p25–p75)	22.7 (20.8–25.8)	23.7 (21.4–26.6)	24.5 (22.0–27.5)	<0.001
Normal weight	532 (68.5)	431 (63.3)	118 (56.5)	
Overweight	163 (21.0)	182 (26.7)	60 (28.7)	0.005
Obese	80 (10.3)	68 (10.0)	31 (14.8)	
Geographical area of birth				0.005
Northern Italy	679 (87.3)	587 (86.2)	173 (82.4)	
Central Italy	27 (3.5)	12 (1.8)	3 (1.4)	
South/Isles	55 (7.1)	64 (9.4)	21 (10.0)	
Abroad	17 (2.2)	18 (2.6)	13 (6.19)	
Education				0.823
Middle school	73 (9.4)	56 (8.2)	15 (7.2)	
High school	275 (35.5)	239 (35.1)	73 (34.9)	
Bachelor’s degree	427 (54.1)	386 (56.7)	121 (57.9)	
Job title				0.173
Nurse	364 (47.0)	299 (43.9)	88 (42.1)	
Physician	100 (12.9)	96 (14.1)	27 (12.9)	
Technician	90 (11.6)	78 (11.4)	36 (17.2)	
Administrative	84 (10.8)	84 (12.3)	31 (14.8)	
Other HW	137 (17.7)	124 (18.2)	27 (12.9)	
Working seniority (Median, p25–p75)	20 (10–28)	19 (10–28)	18 (9–27)	0.485
Hypertension (drug-treated)				0.543
Yes	112 (15.7)	94 (13.8)	29 (13.9)	
No	653 (84.2)	587 (86.2)	180 (86.1)	
Smoking habits				0.017
Non-smoker	550 (71.0)	497 (73.0)	132 (63.2)	
Former smoker	94 (12.1)	94 (13.8)	40 (19.1)	
Smoker	131 (16.9)	90 (12.2)	37 (17.7)	

*p*-values were computed using the Kruskal–Wallis test for quantitative variables and Pearson’s chi-squared for categorical variables, and considered statistically significant if *p* < 0.05.

**Table 3:** Determinants of salt intake evaluated by a multinomial logistic model using adequate salt intake as the reference category. The effects were synthesized through the relative risk ratios (RRRs).

	Moderate Salt Intake		High Salt Intake	
	RRR (CI 95%)	*p* Value	RRR (CI 95%)	*p* Value
Sex				
Female	Ref.		Ref.	
Male	1.5 (1.1–2.0)	0.003	1.3 (0.8–1.9)	0.254
Age per 10 year increase	1 (0.8–1.1)	0.572	0.8 (0.7–0.9)	0.019
BMI				
Normal weight	Ref.		1 (reference)	
Overweight	1.3 (1.0–1.7)	0.051	1.7 (1.2–2.5)	0.004
Obese	1.0 (0.7–1.5)	0.869	1.9 (1.2–3.1)	0.007
Job Title				
Nurse	Ref.		Ref.	
Physician	1 (0.7–1.4)	0.919	1.2 (0.7–2.0)	0.556
Technician	1 (0.7–1.4)	0.878	1.8 (1.1–2.9)	0.015
Administrative	1.2 (0.8–1.7)	0.379	1.7 (1.0–2.8)	0.038
Other	1.3 (0.8–1.5)	0.384	0.8 (0.5–1.4)	0.513
Smoking Habits				
Never	Ref.		Ref.	
Former	1.1 (0.8–1.5)	0.581	1.8 (1.2–2.8)	0.005
Current smokers	0.7 (0.6–1.0)	0.063	1.2 (0.8–1.8)	0.355

**Table 4:** Comparison of answers to the dietary salt intake questionnaire before and after the intervention among 95 health workers who completed the clinical phase of the study.

	Baseline *n* = 95 (%)	Follow-Up *n* = 95 (%)	*p*-Value
How often do you use salt at the table?			<0.001
Never or rarely	34 (34.8)	71 (74.7)	
Quite often	41 (43.2)	23 (24.2)	
Always or very often	20 (21.0)	1 (1.1)	
How much bread do you eat in one day?			<0.001
Usually, bread without or with very little salt	1 (1.1)	33 (34.7)	
Not more than 3 slices a day	71 (74.7)	51 (53.7)	
More than 3 slices a day	23 (24.3)	11 (11.6)	
How many times a week do you eat cheese and/or cold cuts?			<0.001
0–2 times a week	18 (18.9)	48 (50.5)	
3–4 times a week	55 (57.9)	42 (44.2)	
5 or more times a week	22 (23.2)	5 (5.3)	
Do you ever get thirsty, especially after a meal?			<0.001
Never or rarely	40 (42.1)	75 (78.9)	
Quite often	50 (52.6)	19 (20.0)	
Always or very often	5 (5.3)	1 (1.1)	
When you eat out, food usually seems?			<0.001
Salty	8 (8.4)	41 (43.2)	
Normal	79 (83.2)	52 (54.7)	
Insipid	8 (8.4)	2 (2.1)	

*p*-values were computed using Wilcoxon signed-rank test and considered statistically significant if *p* < 0.05.

**Table 5:** Anthropometric indexes and bioelectrical impedance values of the 95 HW who completed the clinical phase, measured before and after the intervention.

	Baseline Median (p25–p75)	Follow-Up Median (p25–p75)	*p*-Value
Weight (kg)	78 (62–87)	75 (62–86)	**<0.001**
BMI	26.1 (24.0–30.1)	25.9 (23.5–29.7)	**<0.001**
Max arterial pressure (mmHg)	130 (120–140)	120 (120–130)	**<0.001**
Min arterial pressure (mmHg)	85 (80–92)	80 (75–80)	**<0.001**
Abdomen circumference (cm)	90 (78–98)	89 (78–98)	**0.018**
Hip circumference (cm)	98 (90–107)	98 (91–106)	0.248
Waist/hip ratio	0.91 (0.87–0.96)	0.91 (0.85–0.95)	**0.028**
Resistance	540.9 (488.8–600)	547.3 (489.8–601.7)	0.153
Reactance	57.5 (50.7–64.2)	56.8 (51.5–62.3)	0.144
Phase angle	6.1 (5.6–6.5)	6 (5.7–6.4)	0.056
Fat-free mass	49.5 (45.1–57.7)	50.2 (44.5–58.9)	**0.036**
Fat mass	23 (17.2–32)	21.5 (16.4–29.4)	0.091

*p*-values were computed using Wilcoxon signed-rank test. Significant results are highlighted in bold.

## Data Availability

The datasets used for this analysis are available on reasonable request by contacting Stefano Porru: stefano.porru@univr.it.

## Supplementary Materials

The following supporting information can be downloaded at: https://www.mdpi.com/article/10.3390/ijerph20085478/s1, Table S1: Sociodemographic characteristics of 350 responders who met the criteria for inclusion in the clinical phase of the study.
