# Supplementary material for: Reduction of Excessive Dietary Sodium Consumption: Effectiveness of a Prevention Intervention among Health Workers in a Large Italian Hospital
Source: Int J Environ Res Public Health. 2023 Apr 12;20(8):5478. doi: 10.3390/ijerph20085478 (PMC10138373; doi:10.3390/ijerph20085478)
Supplement: Supplementary file 1 [file ijerph-20-05478-s001.zip › ijerph-2259372-supplementary.pdf]

Supplementary material

Table S1. Sociodemographic characteristics of 350 responders who met the criteria for inclusion in the clinical phase of the study.

	n=350 (%)
**Sex**	
Female	261 (74.6)
Male	89 (25.4)
**Age** (median, p25-p75)	52 (45-56)
**Height in cm** (median, p25-p75)	166 (160-173)
**Weight in Kg** (median, p25-p75)	74 (62-85)
**BMI** (median, p25-p75)	25.9 (23.1-30.4)
**Geographical area of birth**	
North Italy	294 (84.0)
Center	4 (1.1)
South/Isles	37 (10.6)
Abroad	15 (4.3)
**Education**	
Middle school	36 (10.3)
High school	138 (39.4)
Bachelor's Degree	176 (50.3)
**Job title**	
Nurse	137 (39.1)
Physician	46 (13.1)
Technician	51 (14.6)
Administrative	54 (15.4)
Other HW	62 (17.7)
**Working seniority** (median, p25-p75)	19.5 (10-28)
**Hypertension**	
Yes	123 (35.1)
No	227 (65.9)
**Smoking habits**	
Smoker	49 (14.0)
No smoker	232 (66.3)
Former smoker	69 (19.7)
